# Non-arteritic anterior ischemic and glaucomatous optic neuropathy: Implications for neuroretinal rim remodeling with disease severity

**DOI:** 10.1371/journal.pone.0286007

**Published:** 2023-05-18

**Authors:** Brennan D. Eadie, Oksana M. Dyachok, Jack H. Quach, Charles E. Maxner, Paul E. Rafuse, Lesya M. Shuba, Jayme R. Vianna, Balwantray C. Chauhan, Marcelo T. Nicolela

**Affiliations:** Department of Ophthalmology and Visual Sciences, Dalhousie University, and Nova Scotia Health Authority, Halifax, Canada; Bascom Palmer Eye Institute, UNITED STATES

## Abstract

**Purpose:**

Post-acute non-arteritic ischemic optic neuropathy (NAION) and glaucomatous optic neuropathy (GON) can be difficult to differentiate clinically. Our objective was to identify optical coherence tomography (OCT) parameters to help differentiate these optic neuropathies.

**Methods:**

We compared 12 eyes of 8 patients with NAION and 12 eyes of 12 patients with GON, matched for age and visual field mean deviation (MD). All patients underwent clinical assessment, automated perimetry (Humphrey Field Analyzer II; Carl Zeiss Meditec, Dublin, CA, USA), and OCT imaging (Spectralis OCT2; Heidelberg Engineering, Heidelberg, Germany) of the optic nerve head and macula. We derived the neuroretinal minimum rim width (MRW), peripapillary retinal nerve fibre layer (RNFL) thickness, central anterior lamina cribrosa depth, and macular retinal thickness.

**Results:**

MRW was markedly thicker, both globally and in all sectors, in the NAION group compared to the GON group. There was no significant group difference in RFNL thickness, globally or in any sector, with the exception of the temporal sector that was thinner in the NAION group. The group difference in MRW increased with increasing visual field loss. Other differences observed included lamina cribrosa depth significantly greater in the GON group and significantly thinner central macular retinal layers in the NAION group. The ganglion cell layer was not significantly different between the groups.

**Conclusions:**

The neuroretinal rim is altered in a dissimilar manner in NAION and GON and MRW is a clinically useful index for differentiating these two neuropathies. The fact that the difference in MRW between the two groups increased with disease severity suggests distinct remodelling patterns in response to differing insults with NAION and GON.

## Introduction

Glaucoma, the most common cause of irreversible vision loss worldwide, is a multifactorial, progressive, degenerative optic neuropathy that can lead to blindness if untreated [1]. Accurate and early diagnosis of glaucoma is critical for reducing the likelihood of visual disability from glaucoma and identifying other forms of optic neuropathy that may necessitate distinct medical management [2–4]. Classically, characteristic abnormalities in the clinical appearance of the optic disc have been viewed as central to the diagnosis of glaucomatous optic neuropathy (GON) [5, 6]. With the advent of optical coherence tomography (OCT), [7–10] clinicians now have access to a large amount of data on various structural parameters related to the optic nerve head (ONH), peripapillary retina, and macula that hold significant potential for improved diagnostic accuracy of GON [11–13].

Non-glaucomatous optic neuropathies that are potentially confused with GON include ischemic optic neuropathy, compressive optic neuropathy, and congenital optic disc defects [14]. The non-glaucomatous optic neuropathies that are most frequently confused for GON are the ischemic optic neuropathies, most notably Non-arteritic Anterior Ischemic Optic Neuropathy (NAION) in the post-acute phase of the disease [14]. In the acute phase, NAION can be distinguished relatively easily from GON by characteristic disc swelling, hyperemia, and hemorrhage; however, diagnostic confusion can exist in post-acute phases, particularly in the context of an unclear history. Both post-acute NAION and GON can lead to the appearance of optic disc excavation and both entities can lead to altitudinal visual field defects [15].

The objective of the current study was to identify which OCT parameters are most valuable in distinguishing GON from post-acute NAION. These data could help provide clinicians with practical evidence-based methods to assist in the interpretation of OCT data when attempting to distinguish GON from post-acute NAION.

## Materials and methods

### Subjects

Subjects with a confirmed diagnosis of NAION that occurred more than 1 year (post-acute) prior to assessment were recruited from Nova Scotia Health Authority glaucoma and neuro-ophthalmology subspecialty clinics of authors MN, LS, PR, and CM. The study was approved by the Nova Scotia Health Authority Research Ethics Board. In accordance with the Declaration of Helsinki, all subjects gave informed consent to participate.

Inclusion criteria for subjects were: (i) age between 18 and 90 years, (ii) a diagnosis of NAION confirmed by a neuro-ophthalmologist (CM) and (iii) refractive error ≤ 6D spherical equivalent and ≤ 2D of astigmatism. Subjects were excluded if any of the following were found: (i) ocular pathology other than NAION, (ii) previous ocular surgery (excluding cataract or refractive surgery), or (iii) inability to provide informed consent.

A verbal screening for willingness to be contacted for participation was first conducted. Patients were subsequently contacted by telephone and recruited for the study. At the study visit, the protocol was reviewed with the patient and informed consent obtained. Patients then underwent a full clinical assessment, automated perimetry, and OCT imaging. Automated perimetry was performed using the Swedish Interactive Thresholding Algorithm (SITA) Standard thresholding strategy, 24–2 program of the Humphrey Field Analyzer II (Carl Zeiss Meditec, Dublin, CA, USA).

Eyes of patients with NAION were matched to eyes of patients with GON based on age and visual field mean deviation (MD). The matched GON patients were obtained in a sequential, retrospective manner from the glaucoma research database at our centre and underwent the same testing protocol as the NAION patients within 2 years prior to cessation of NAION data collection with a best effort made to match patients as closely as possible for both visual field mean deviation and age.

### OCT imaging

Imaging was performed with a commercially available device (Spectralis OCT2; Heidelberg Engineering, Heidelberg, Germany) and scans of the ONH, peripapillary retina, and macula were obtained. Image acquisition and analysis was performed in a consistent orientation according to each subject’s fovea to Bruch’s membrane opening axis (Anatomical Positioning System, Heidelberg Engineering). All segmentations were manually checked and corrected if necessary. The ONH scan comprised 24 B-scans in a radial pattern centred on Bruch’s membrane opening (BMO) and yielded measurements of the neuroretinal minimum rim width (MRW) and BMO area. Radial scans were also used to determine central lamina cribrosa (LC) depth from the anterior laminar surface. A scientific version of Spectralis software (Spectralis Viewing Module version 6.4.8.114) was used to obtain the LC depth. As described in detail elsewhere, [16] LC depth was obtained by centring a peripapillary ring on the BMO (inner radius 1700 μm; outer radius 1800 μm) and manually segmenting the anterior LC and anterior sclera in all radial scans. The position of the anterior sclera within the peripapillary ring was used to create a reference plane (3D best fit of all radial scans). The anterior lamina cribrosa surface between the radial scans was interpolated with a cubic spline. The vertical distance between the reference plane and the anterior lamina surface was measured at each pixel of the surface and the average of these vertical measurements constituted the LC depth [17].

The peripapillary retinal nerve fibre layer (RNFL) thickness was obtained from a 3.5 mm diameter peripapillary circular scan centred on the BMO. Importantly, due to the image acquisition positioned according to the FOBMO (Fovea to Bruch’s Membrane Opening) angle, both the MRW and RNFL data are precisely concordant relative to orientation. The macula was imaged using 61 B-scans in a raster pattern centred on the fovea and parallel to the FOBMO angle. Thickness values of the macular RNFL, ganglion cell layer, inner plexiform layer, inner nuclear layer, outer plexiform layer, and outer nuclear layer were obtained.

MRW and peripapillary RNFL measurements are classified as being borderline if below the 5^th^ percentile and outside normal limits if below the 1^st^ percentile with respect to the normative databases.

### Data analyses

The global and 6 sectoral MRW and peripapillary RNFL values for each pair of NAION and GON eyes were compared. The retinal layer thickness values were compared within macular retinal regions according to the Early Treatment of Diabetic Retinopathy Study (ETDRS) protocol [17]. Comparisons were made between groups for matching altitudinal defects (*e*.*g*., superior, inferior, or both superior and inferior visual field loss).

Group variables were reported as medians with interquartile ranges (IQR). Group comparisons of continuous variables were made with the non-parametric Wilcoxon tests and group comparisons of categorical variables were made with the chi-squared test. To determine if OCT differences between NAION and GON changed with disease severity, Pearson correlation coefficients were calculated between visual field MD and the OCT parameters: (i) global peripapillary RNFL, (ii) global MRW, (iii) lamina cribrosa depth, and (iv) central macular retinal layers. Statistical differences between correlations were calculated using Fisher *r* to *z* transformations. Bonferroni corrections were used for multiple comparisons. Statistical significance was assumed at an alpha value of 0.05. All analyses were performed with commercial software (SPSS, version 26 for Macintosh, IBM, Armonk, NY).

### Results

There were 12 eyes of 8 patients with NAION and 12 eyes of 12 patients with GON in the study. There were 4 males and 4 females, with median age 70 years, in the NAION group and 4 males and 8 females, with median age 67 years, in the GON group. We accepted a difference of 7 years in age and 3.2 dB (<+/-15%) visual field mean deviation (MD) between the paired NAION and GON eyes.

### Clinical assessment

The clinical characteristics of the patients are shown in Table 1. There was a trend towards significantly worse logMAR best corrected visual acuity in the NAION group of 0.44 (0.12 to 1.27) compared to the GON group of 0.18 (IQR 0.10 to 0.27; *P* = 0.08). The treated IOP in the GON group of 16.5 (10.25 to 20.0) was not significantly different to that of the NAION group of 13.0 (IQR 11.25 to 14.75; *P* = 0.37).

**Table 1:** Demographic and clinical characteristics of patients with NAION and their paired GON patient by age and visual field mean deviation.

Patient / Eye	Diagnosis	Gender	BCVA	MD	IOP
**1 / R**	NAION	F	20/25 (0.10)	-2.58	14
**10 / L**	GON	F	20/25 (0.10)	-2.42	16
**2 / R**	NAION	M	20/30 (0.18)	-13.27	15
**11 / L**	GON	M	20/25 (0.10)	-12.64	9
**3 / R**	NAION	F	20/300 (1.18)	-13.89	13
**12 / L**	GON	M	20/40 (0.30)	-12.2	9
**4 / R**	NAION	M	20/20 (0.00)	-8.96	11
**13 / R**	GON	F	20/40 (0.30)	-9.00	14
**5 / R**	NAION	M	20/20 (0.00)	-19.82	21
**14 / R**	GON	F	20/30 (0.18)	-18.09	15
**6 / L**	NAION	M	20/400 (1.30)	-23.49	16
**15 / R**	GON	M	20/30 (0.18)	-24.20	21
**7 / R**	NAION	F	20/400 (1.30)	-28.07	10
**16 / L**	GON	F	20/20 (0.00)	-25.61	18
**7 / L**	NAION	F	20/60 (0.48)	-26.26	10
**17/ L**	GON	M	20/20 (0.00)	-23.39	20
**8 / R**	NAION	M	20/100 (0.70)	-25.06	12
**18 / R**	GON	F	20/200 (1.00)	-27.86	4
**8 / L**	NAION	M	CF 2ft (2.00)	-30.69	12
**19 / L**	GON	M	20/30 (0.18)	-27.62	20
**9 / R**	NAION	F	20/40 (0.30)	-11.92	13
**20 / L**	GON	M	20/25 (0.10)	-13.70	17
**9 / L**	NAION	F	20/50 (0.40)	-22.88	14
**21 / L**	GON	M	20/30 (0.18)	-19.75	20

NAION = Non-arteritic Anterior Ischemic Optic Neuropathy; GON = Glaucomatous Optic Neuropathy; R = Right; L = Left; MD = Mean Deviation; Gender F = Female and M = Male; BCVA = Best Corrected Visual Acuity (logMAR); IOP = Intraocular Pressure.

### Visual field defects

There was a wide range of severity of visual field loss. The median MD in the NAION and GON groups was -21.35 (-12.25 to -25.96) and -18.92 (-12.31 to -25.26), respectively. Ten eyes in the NAION group and 8 eyes in the GON group had visual field defects in both superior and inferior hemifields.

### OCT parameters

**Fig 1:**
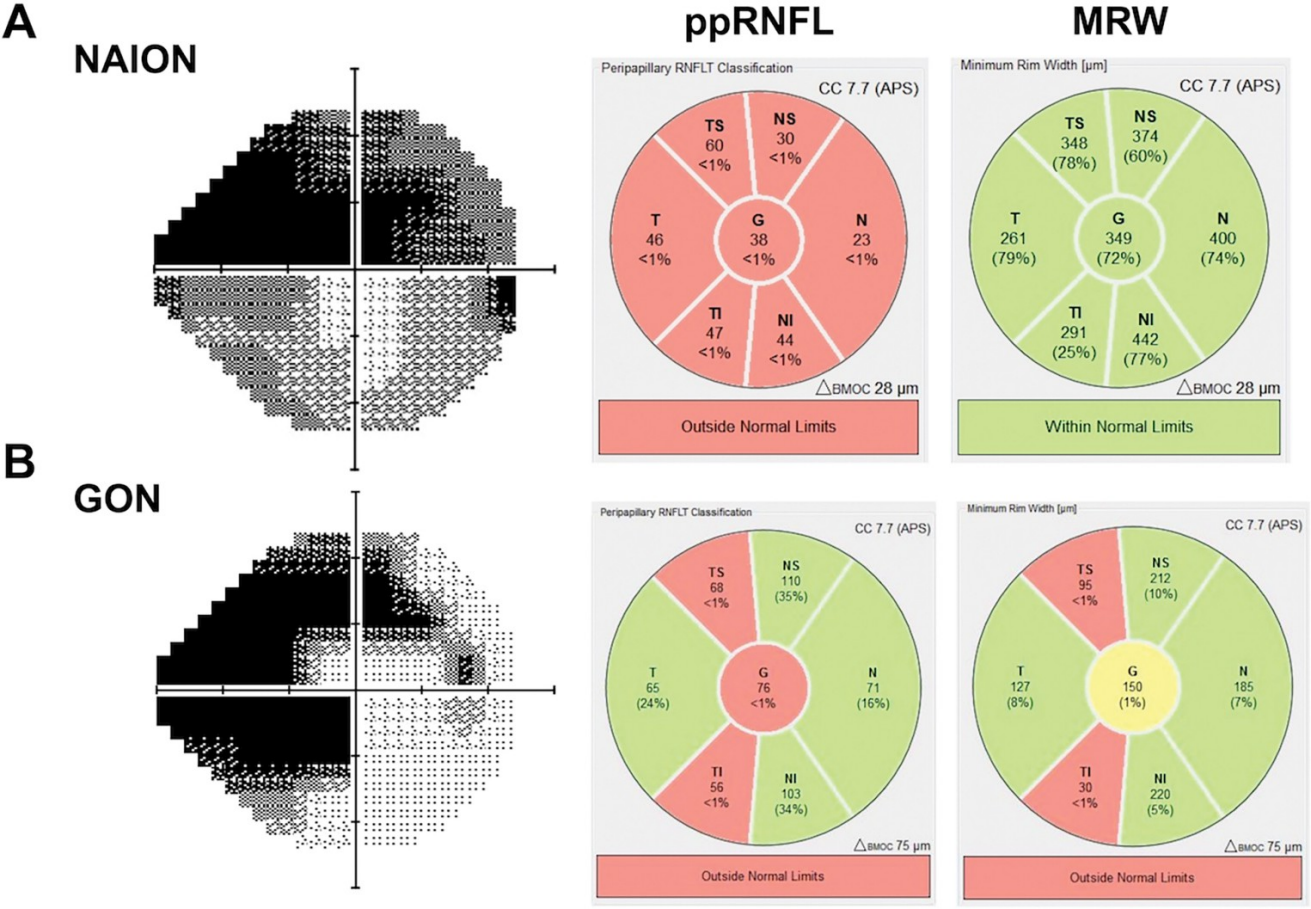
Peripapillary RNFL and MRW in a NAION patient compared to GON. Both patients had prominent superior greater than inferior visual field loss (greyscale; left). The peripapillary retinal nerve fibre layer (RNFL) thinning is prominent diffusely in the NAION case including temporally, whereas the thinning in the GON case is preferentially superotemporal and inferotemporal. The minimum rim width (MRW) grossly matches the peripapillary RNFL in the GON case. In contrast, the MRW is not thin, and is in fact, above average in the NAION case.

The results for OCT parameters are shown in Table 2. The BMO area was not significantly different between the NAION and GON groups (NAION: median = 1.91 mm^2^; IQR, 1.75 to 2.10 mm^2^; GON: median = 1.85 mm^2^; IQR, 1.55 to 2.14 mm^2^; *P* = 0.93). The global peripapillary RNFL was not significantly different between eyes with NAION and GON. The peripapillary RNFL was outside normal limits for the majority of eyes in both groups (10 eyes with NAION and 11 eyes with GON; *P* = 0.59). Although globally there were no differences in the peripapillary RNFL thickness, the temporal peripapillary RNFL was significantly thinner in the NAION group (*P*<0.025). In contrast to the peripapillary RNFL analyses, the global MRW was thicker in the NAION group versus the GON group (*P*<0.005). Global MRW was outside normal limits in only 1 eye with NAION compared to 9 eyes with GON (*P*<0.001). MRW was thicker in all 6 sectors in the NAION group (*P*<0.005). A pair of cases, one from each group, with similar MD showing significantly different degrees of peripapillary RNFL and MRW loss is shown in Fig 1.

**Table 2:** Global and sectoral peripapillary RNFL and MRW differences between NAION and GON patients.

		NAION (median ± IQR)	GON (median ± IQR)	*P*
**Peripapillary RNFL (F06Dm)**				
**Global**		51 (42–58)	60 (45–71)	0.36
**Sectors**	**ST**	60 (54–71)	62 (38–73)	0.44
	**SN**	53 (41–74)	63 (47–87)	0.15
	**N**	38 (32–44)	57 (45–67)	0.13
	**IN**	68 (46–93)	62 (47–76)	0.49
	**IT**	67 (54–85)	65 (45–94)	0.27
	**T**	39 (33–46)	51 (38–62)	**<0.025**
**MRW (F06Dm)**				
**Global**		317 (260–347)	151 (134–172)	**<0.005**
**Sectors**	**ST**	284 (202–317)	78 (54–136)	**<0.005**
	**SN**	354 (258–383)	155 (116–201)	**<0.005**
	**N**	354 (292–386)	193 (143–221)	**<0.005**
	**IN**	390 (343–443)	197 (142–236)	**<0.005**
	**IT**	296 (240–350)	136 (106–159)	**<0.0005**
	**T**	241 (185–249)	125 (106–141)	**<0.005**

NAION = Non-arteritic Anterior Ischemic Optic Neuropathy; GON = Glaucomatous Optic Neuropathy; RNFL = Retinal Nerve Fibre Layer; MRW = Minimum Rim Width; ST = Superotemporal; SN = Superonasal; N = Nasal; IN = Inferonasal; IT = Inferotemporal; T = Temporal.

### Lamina cribrosa depth

**Fig 2:**
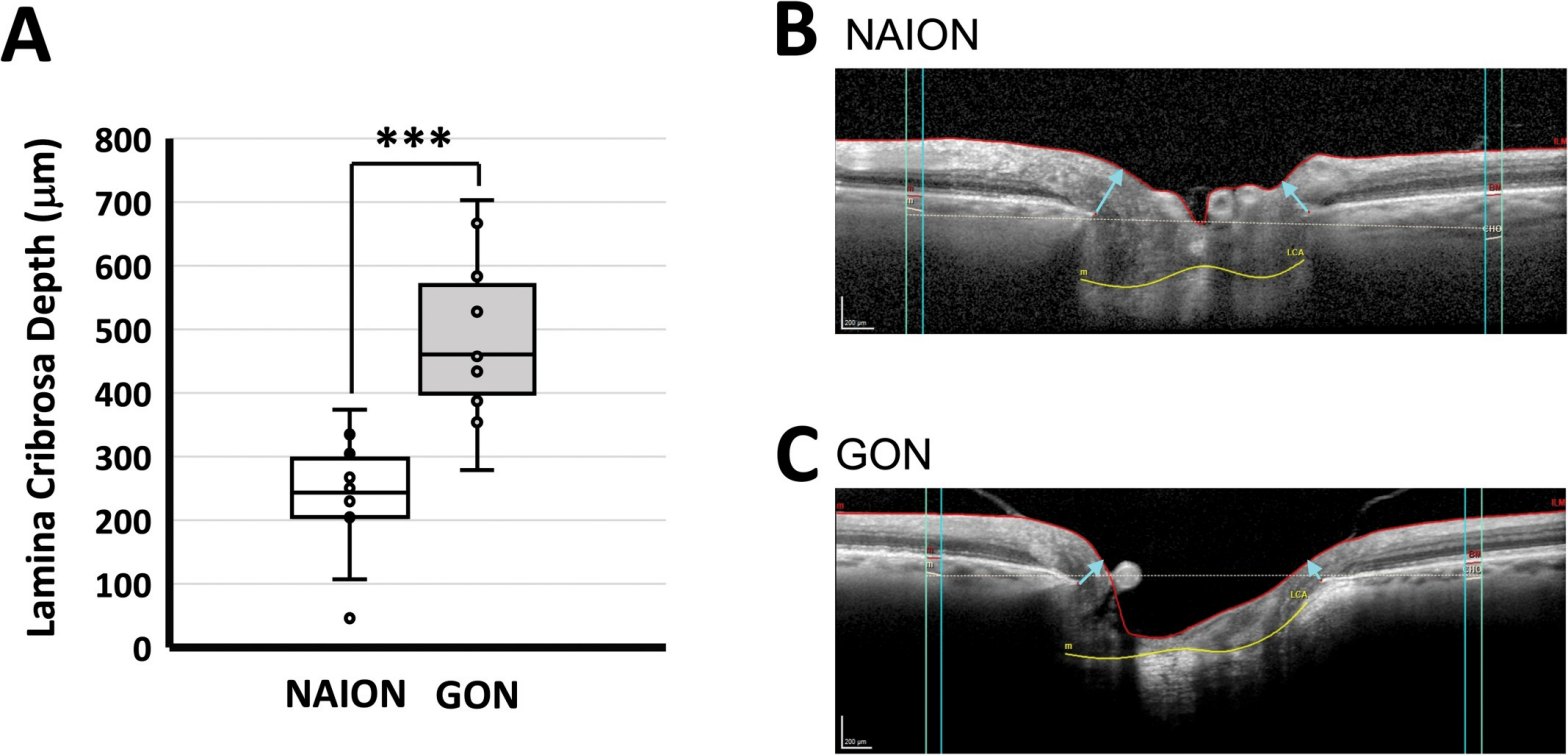
Lamina cribrosa depth. (A) The lamina cribrosa depth (μm) was significantly greater in the GON (Glaucomatous Optic Neuropathy) group than the NAION (Non-Arteritic Anterior Ischemic Optic Neuropathy) group. An example pair of cases showing greater lamina cribrosa depth in the NAION (B) compared to the GON (C). The difference between the dashed line and yellow line (anterior lamina cribrosa) is the lamina cribrosa depth. Cyan arrows indicated the minimum rim width (MRW) measurement. The ILM is demarcated by the red line. Box and whisker plots show individual data, median, and interquartile ranges. ***denotes P < 0.001.

The median LC depth was significantly shallower in eyes with NAION (244.08 μm; 205.06 to 297.66) compared to eyes with GON (461.18 μm; 399.43 to 569.91; *P*<0.01; Fig 2A). Illustrative cases are shown in Fig 2B & 2C.

### Macular retinal sectors and layers

**Fig 3:**
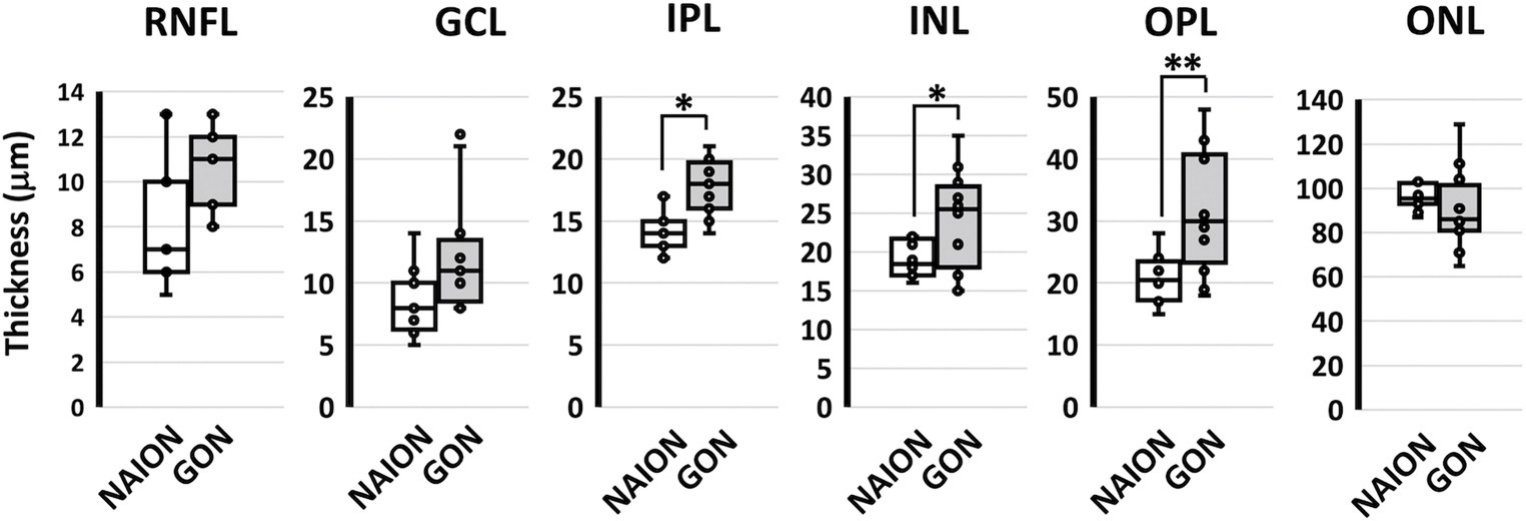
Thickness of central retinal layers. The central retinal layers were thinner in the NAION (Non-arteritic Anterior Ischemic Optic Neuropathy) group compared to the GON (Glaucomatous Optic Neuropathy) group for the inner plexiform layer (IPL), inner nuclear layer (INL), and outer plexiform layer (OPL). There was no difference in the retinal nerve fibre layer (RNFL), ganglion cell layer (GCL), or outer nuclear layer (ONL). Box and whisker plots show individual data, median, and interquartile ranges. Correction was made for multiple comparisons using the Bonferroni method. *denotes P < 0.05 and **denotes P < 0.01.

The thickness of the macula was not significantly different between NAION and GON groups. Significant image artifact was observed for one eye of one patient in the NAION group and these data were excluded from analysis. After exploratory subregion analysis, subregions found to be significantly thinner in the NAION group compared to the GON group were the central inner plexiform layer (IPL; *P*<0.05), central inner nuclear layer (INL; *P*<0.05), central outer plexiform layer (OPL; *P*<0.01; Fig 3). The GCL was not significantly different between groups for any subregions (*P*>0.025).

### Correlation to severity of visual field loss

**Fig 4:**
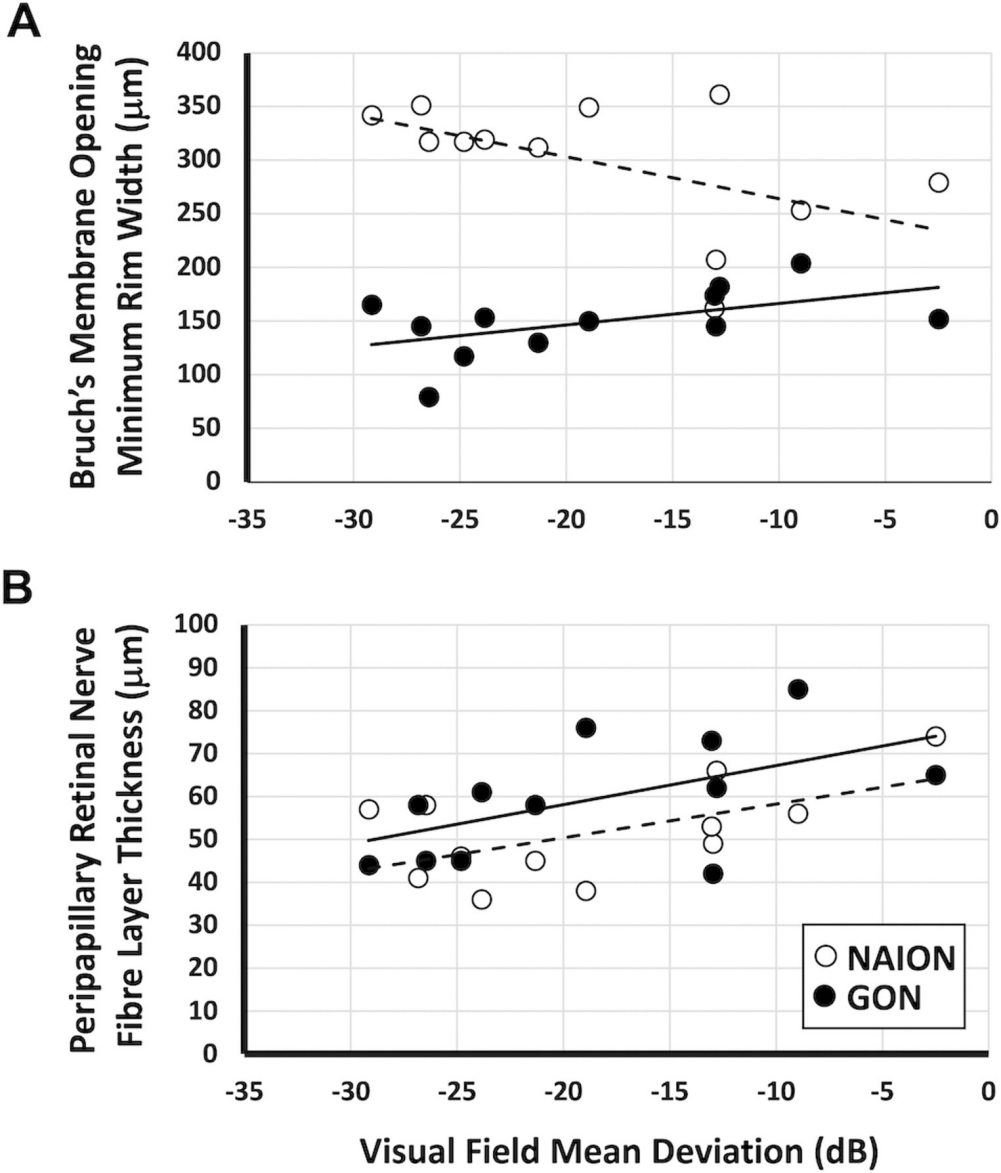
Correlations of peripapillary RNFL and MRW thickness with visual field mean deviation. (A) NAION and GON show diverging correlations with regards to minimum rim width (MRW). A more obvious separation occurs at more negative visual field mean deviations. (B) Similar positive correlation between peripapillary retinal nerve fibre layer (RNFL) thickness and visual field mean deviation for both the NAION and GON groups.

In the NAION group, MRW increased with worse MD (*r* = -0.53; *P*<0.05). In contrast, the MRW in the GON group decreased with disease severity (*r* = 0.52; *P*<0.05; Fig 4A). This dissociation in the direction of the correlation with visual field loss was not evident with the peripapillary RNFL which showed a similar decrease with worse MD in both the NAION (*r* = -0.58) and the GON (*r* = -0.55) groups (*P* = 0.99; Fig 4B). There was no significant correlation observed between the lamina cribrosa depth and severity of visual field loss (*P* = 0.26). There were also no significant correlations between severity of visual field loss and the central macular retinal subregions identified as different between groups (IPL: *P* = 0.15; INL: *P* = 0.20; OPL: *P* = 0.26).

## Discussion

In this study, we evaluated a variety of OCT parameters in eyes with NAION and GON, matched for both age and visual field damage. The most notable difference observed with OCT was the minimum rim width (MRW) that was markedly different between groups globally and in all sectors. This was in contrast to the peripapillary RNFL analysis, the metric most commonly used for assessment of GON, which did not show a difference either globally or in 5 of the 6 sectors. Interestingly, MRW correlated with visual field damage for both NAION and GON but did so in opposite directions; MRW increased with disease severity in NAION eyes and decreased with disease severity in GON eyes. Consistent with previous studies, LC depth was greater in GON than NAION eyes. Exploratory analyses suggested potential differences in the central macular retinal subregions. Unlike MRW, these differences were not increasingly apparent with increasing visual field damage.

Previous studies have shown that GON is associated with a thinning of the MRW; whereas, in NAION the MRW appears to be spared [18, 19]. For example, one recent study has suggested that optic neuropathies other than glaucoma, including NAION, may be distinguished by an MRW that is within normal limits compared to thinning of the MRW observed in patients with glaucoma and a history of normal IOPs [20]. In another recent study, NAION patients and healthy subjects had similar MRW in all sectors, whereas glaucoma patients had significantly thinner MRW [21]. In this latter study, the peripapillary RNFL was not significantly different between the NAION and GON groups in all sectors. Our findings generally support previous findings; however, we did observe significantly greater peripapillary RNFL thinning temporally in the NAION group compared to the GON group. Our observation is consistent with the hypothesis of NAION pathophysiology involving hypo- or non-perfusion of the posterior ciliary arteries to the optic nerve head and a temporal watershed zone [15, 22]. Lack of perfusion to the optic nerve head in NAION may be more likely to cause ischemia in distal regions of the arterioles which includes the temporal optic nerve head. This observation of temporal peripapillary RNFL thinning is also consistent with the clinical observation of tendency towards worse visual acuity, and perhaps our observation of thinner central macular retinal subregions, in the NAION group.

A novel finding in our study was the striking difference between MRW and peripapillary RNFL thickness as the degree of visual field damage increases. This extends upon previous studies that have simply noted differences in the MRW between NAION and GON. Specifically, we found that with more visual field damage, the MRW was increasingly thicker in NAION eyes and the MRW was increasingly thinner in GON eyes. The clinical relevance of this dissociation is important. As glaucoma progresses, the near complete loss of prelaminar neuroretinal rim tissue and exposure of the lamina cribrosa can be more easily confused with optic disc pallor characteristic of optic neuropathies other than GON, such as NAION. This confusion is less likely to occur in cases of mild or moderate concentric neuroretinal rim excavation.

The anatomical basis for decreasing MRW with disease severity is well known for GON and represents one consequence of optic nerve head remodeling: prelaminar tissue loss [23–25]. Similarly, the anatomical basis for increased lamina cribrosa depth is well known for GON and represents the other consequence of optic nerve head remodeling: excavation and posterior dislocation of the lamina cribrosa. Prelaminar and laminar remodeling occur in GON in response to a complex interaction between biomechanical, vascular, and cellular factors [23, 26]. In contrast, in NAION, the insult is clearly an acute vascular event. Swelling and hypermia of the optic disc are characteristic clinical features in the acute phase, whereas pallor of the neuroretinal rim is characteristic of the post-acute NAION. Interestingly, our data suggest that optic nerve head remodeling may also occur in post-acute NAION as we observed an increasing MRW with disease severity. Perhaps this should not be surprising, considering the observations that remodeling in GON may occur not only in response to mechanical factors (*e*.*g*., translaminar pressure gradient), but also to metabolic stress, inflammation, and glial cell reactivity [26]. These findings lead to questions on the nature of remodeling over the long-term in NAION. Although studies on this topic are sparse, the anatomical basis for the increasingly apparent MRW dissociation may be disorganization and loss of glioarchitecture in GON versus hypertrophy of the glioarchitecture in NAION [27]. These opposing remodeling processes may become increasingly obvious with more severe insults to the optic nerve head and greater visual field damage.

The increasing dissociation of GON and NAION with disease severity seen with the MRW was not observed for any other OCT parameter. However, consistent with previous reports, our data did show that the LC depth is also a distinguishing factor between NAION and GON in general [28, 29]. There was no relationship observed between LC depth and severity of visual field damage in either group. It is possible that in the glaucoma group, this relationship was not significant because of the relatively advanced damage in these patients as well as the small sample size. Interestingly, in NAION, the LC may be more anterior relative to healthy subjects prior to insult, but deepen with the acute insult, and then reverse to a relatively normal position [30]. Unfortunately, LC depth is not currently included as an automated output with commercially available OCT software.

Ganglion cell layer/complex (GCL/GCC) on the macular retinal OCT has become increasingly appreciated as an important part of the assessment of glaucomatous optic neuropathy [31, 32]. Ganglion cell complex thinning has been observed in both NAION and GON relative to controls [33]. Our exploratory analyses did not reveal differences in the GCL/GCC; however, the central macular retinal subregions inner plexiform layer, inner nuclear layer, and outer plexiform layers were thinner in the NAION group versus the GON group, consistent with the overall poorer visual acuity in NAION than the GON group and general clinical experience [34]. These exploratory analyses suggest that subregion analysis of the central macula may be useful adjunctive information in distinguishing NAION and GON. We did not observe significant correlations between central macular retinal subregion thickness to severity of visual field damage.

The strengths of the current study relative to previous reports utilizing OCT to evaluate NAION and GON include the pairwise matching for visual field damage with a range of visual field defects in a typical clinical population, the use of granular information from a variety of OCT parameters, and the correlation of OCT differences with disease severity as indicated by visual field damage. Magnitude of these differences in a small sample size highlights the clinical relevance of these observations; however, the small sample size is a weakness in that more subtle structural differences that exist may not have been identified.

In conclusion, the MRW was the OCT parameter that most clearly could distinguish between GON and NAION. Further, the dissociation of NAION from GON with MRW appears to become increasingly obvious with greater disease severity. This is clinically relevant as the most difficult cases to distinguish may be the most severe. The pathogenesis of the increasing MRW difference may be remodeling via the loss of neural tissue and glioarchitecture in GON versus hypertrophy of the glioarchitecture in NAION. Future studies should longitudinally investigate the unique remodeling of the prelaminar optic nerve head, and the time course of this remodeling, in these two common optic neuropathies.

## Supporting information

S1 File All relevant data are within this file. (XLSX)
